# 

**Published:** 1930

**Authors:** 


### CONTENTS.

| | Page |
|---|---|
| **Acute Intestinal Obstruction.** By D. P. D. Wilkie, O.B.E., M.D., Ch.M., F.R.C.S., F.R.S.E. | 97 |
| **Child Guidance—** | |
| I. **Work in New York.** By Mrs. Posthuma, M.B., Ch.B. | 111 |
| II. **Clinics from the Brain Physiologist's Viewpoint.** By R. J. A. Berry, M.D., F.R.C.S., F.R.S.E. | 120 |
| III. **Work in Great Britain.** By R. G. Gordon, M.D., D.Sc., F.R.C.P.E. | 126 |
| **Stokes-Adams Syndrome produced by Digitalis in a Case of Auricular Fibrillation.** By T. F. R. Hewer, M.D. | 135 |
| **Dental Infections: their Diagnosis and Treatment.** By G. F. Fawn, M.R.C.S., L.R.C.P., B.D.S., L.D.S. | 137 |
| **Recent Dental Research.** By R. H. McKeag, M.A., M.B., B.Ch., B.A.O., B.Dent.Sc. | 143 |
| **Reviews of Books** | 149 |
| **Editorial Notes** | 159 |
| **Obituary:** Thomas Moravian Carter, O.B.E., M.D. | 164 |
| **Meetings of Societies** | 165 |
| **The Medical Library of the University of Bristol** | 166 |
| **Local Medical Notes** | 167 |

